# ^18^F-FDG-PET/CT-measured parameters as potential predictors of residual disease after neoadjuvant chemoradiotherapy in patients with esophageal carcinoma

**DOI:** 10.1590/0100-3984.2021.0135

**Published:** 2022

**Authors:** Francisco Tustumi, David Gutiérrez Albenda, Rubens Antonio Aissar Sallum, Sergio Carlos Nahas, Ulysses Ribeiro Junior, Carlos Alberto Buchpiguel, Ivan Cecconello, Paulo Schiavom Duarte

**Affiliations:** 1 Department of Gastroenterology, Digestive Surgery Division, Faculdade de Medicina da Universidade de São Paulo (FMUSP), São Paulo, SP, Brazil.; 2 Department of Radiology and Oncology, Nuclear Medicine Division, Faculdade de Medicina da Universidade de São Paulo (FMUSP), São Paulo, SP, Brazil.

**Keywords:** Esophageal neoplasms, Neoadjuvant therapy, Positron-emission tomography, Nuclear medicine, Neoplasias esofágicas, Terapia neoadjuvante, Tomografia por emissão de pósitrons, Medicina nuclear

## Abstract

**Objective:**

To evaluate the maximum and mean standardized uptake values, together with the metabolic tumor value and the total lesion glycolysis, at the primary tumor site, as determined by ^18^F-fluorodeoxyglucose positron-emission tomography/computed tomography (^18^F-FDG-PET/CT), performed before and after neoadjuvant chemoradiotherapy (nCRT), as predictors of residual disease (RD) in patients with esophageal cancer.

**Materials and Methods:**

The standardized uptake values and the volumetric parameters (metabolic tumor value and total lesion glycolysis) were determined by ^18^F-FDG-PET/CT to identify RD in 39 patients before and after nCRT for esophageal carcinoma. We used receiver operating characteristic curves to analyze the diagnostic performance of ^18^F-FDG-PET/CT parameters in the definition of RD. The standard of reference was histopathological analysis of the surgical specimen.

**Results:**

Eighteen patients (46%) presented RD after nCRT. Statistically significant areas under the curve (approximately 0.72) for predicting RD were obtained for all four of the variables evaluated after nCRT. Considering the presence of visually detectable uptake (higher than the background level) at the primary tumor site after nCRT as a positive result, we achieved a sensitivity of 94% and a specificity of 48% for the detection of RD.

**Conclusion:**

The use of ^18^F-FDG-PET/CT can facilitate the detection of RD after nCRT in patients with esophageal cancer.

## Abstract (pt)

**Objetivo:**

Avaliar os valores máximo e médio de captação padronizada, o valor metabólico do tumor e a glicólise total da lesão do local do tumor primário, medidos no estudo de ^18^F-FDG-PET/CT realizado antes e depois da quimiorradioterapia neoadjuvante (nQRT) em pacientes com câncer de esôfago, como preditores de doença residual (DR).

**Materiais e Métodos:**

Os valores máximo e médio de captação padronizada e os parâmetros volumétricos (valor metabólico do tumor e glicólise total da lesão) da ^18^F-FDG-PET/CT realizada em 39 pacientes antes e após a nQRT para carcinoma de esôfago foram avaliados para RD. Usamos curvas *receiver operating characteristic* (ROC) para analisar o desempenho diagnóstico dos parâmetros ^18^F-FDG-PET/CT na definição de RD. O estudo anatomopatológico foi utilizado como padrão ouro.

**Resultados:**

Dezoito pacientes (46%) apresentaram DR após a nQRT. Áreas estatisticamente significativas sob a curva ROC para predizer DR foram obtidas para as quatro variáveis nos estudos realizados após a nQRT, com áreas sob a curva ROC semelhantes em torno de 0,72. Considerando a presença de captação visualmente detectável (captação maior que o *background*) no local da lesão primária após a nQRT como resultado positivo, teríamos uma sensibilidade de 94% e uma especificidade de 48% para detecção de DR.

**Conclusão:**

A ^18^F-FDG-PET/CT pode ser útil para detectar a presença de doença neoplásica residual no câncer de esôfago após a nQRT.

## INTRODUCTION

In locally advanced resectable esophageal cancer, neoadjuvant chemoradiotherapy (nCRT) is currently the standard of care^(1,2)^. After nCRT, pathological regression is the main prognostic indicator for esophageal cancer^(3)^. Currently, patients with esophageal cancer undergo surgical resection of the primary lesion after nCRT regardless of the pathological status of the lesion after the therapy. However, for patients with other types of cancer, such as rectal cancer^(4)^, a watchful waiting approach is often adopted if clinical staging suggests a pathological complete response (pCR) after nCRT. Therefore, if a diagnostic imaging method could accurately identify a pCR after nCRT in patients with esophageal cancer, it would also be possible to identify patients who are the best candidates for a watchful waiting approach, somewhat similar to what is done in cases of rectal cancer^(4)^, thus avoiding the postoperative complications and high mortality associated with esophagectomy.

Functional imaging, such as ^18^F-fluorodeoxyglucose positron-emission tomography/computed tomography (^18^F- FDG-PET/CT), evaluates metabolic activity and may improve patient selection for further treatment^(5)^. There have been studies demonstrating the prognostic value of metabolic tumor volume (MTV) and total lesion glycolysis (TLG) for esophageal cancer treated with trimodal therapy^(6-13)^. However, the use of the ^18^F-FDG-PET/CT to assess the presence of residual disease (RD) after nCRT in patients with esophageal cancer has not been widely studied. In lung and rectal cancer, serial ^18^F-FDG-PET/CT after neoadjuvant therapy can identify predictors of a pathological response^(14,15)^. However, studies using ^18^F-FDG-PET/CT after nCRT in patients with esophageal cancer have produced heterogeneous results. Heneghan et al.^(16)^ evaluated the accuracy of the post-nCRT maximum standardized uptake value (SUV_max_) in detecting a pCR and found it to have a sensitivity of only 56%. McLoughlin et al.^(17)^ showed that ^18^F-FDG-PET/CT had a sensitivity of 62% and specificity of 44% for the detection of a pCR after nCRT. Gabrielson et al.^(18)^ reported significantly greater SUV changes in nCRT responders than in nCRT nonresponders. However, the authors found no significant difference in SUV between the patients with a pCR and those with a subtotal response. In addition, Elliott et al.^(19)^ evaluated esophageal adenocarcinoma only and showed that ^18^F-FDG-PET/CT had poor discriminatory value for clinical application. However, those studies did not evaluate the volumetric parameters determined by ^18^F-FDG-PET/CT and used distinct nCRT regimens. Therefore, our study aims to evaluate the potential of the SUV_max_, mean SUV (SUV_mean_), MTV, and TLG measured at the site of primary esophageal tumor on ^18^F-FDG-PET/CT performed before and after nCRT with a platinum- and taxane-based regimen, as well as the change in those values between the two time points and their association with RD.

## MATERIALS AND METHODS

### Patients and study design

This was a retrospective cross-sectional study of patients with esophageal carcinoma, in which we attempted to determine whether the SUV_max_, SUV_mean_, and volumetric parameters (MTV and TLG) obtained by ^18^F-FDG-PET/CT, before and after nCRT, are associated with the pathological response. We recruited patients from among those under treatment at a single institute between 2009 and 2019. We included 39 patients who had completed a (platinum- and taxane-based) nCRT regimen, followed by esophagectomy with curative intent, and had undergone ^18^F-FDG-PET/CT at least twice: before nCRT; and between the end of nCRT and the esophagectomy. The nCRT included the administration of carboplatin or cisplatin concurrent with radiation (41.4, 45.0, or 50.4 cGy).

The patients were staged with endoscopy, CT, and ^18^F-FDG-PET/CT, after which they were classified according to the 8th edition of the Union for International Cancer Control staging system^(20)^. The local research ethics committee approved the study (Reference no. 1492/19).

An experienced pathologist, blinded to the ^18^F-FDG-PET/CT findings, evaluated the surgical specimens. A pCR to nCRT was defined as no residual malignant cells detected by hematoxylin and eosin staining in the surgical specimen. We defined RD as the presence of any cancer cells (single cells or cell clusters).

### ^18^F-FDG-PET/CT acquisition and imaging analyses

A whole-body PET/CT system with time-of-flight capability (Discovery 690; GE Healthcare, Waukesha, WI, USA) was used for the ^18^F-FDG-PET/CT acquisition. The patients were instructed to fast for at least six hours and were required to have a blood glucose level ≤ 180 mg/dL before injection of the ^18^FDG (≤ 3.7 MBq/kg of body weight). Image acquisition was initiated approximately 60 min after injection of the radiotracer, and images were acquired from the mid-skull to the mid-thigh. The metabolic activity at the primary tumor site, before and after nCRT, was recorded by using the following parameters: SUV_max_, SUV_mean_, TLG, and MTV. Those values were calculated by a single nuclear medicine physician using a radiology workstation (AW VolumeShare 5; GE Healthcare). The SUV thresholds used in order to define the boundaries of the lesions were established by visual analysis. The total volume of interest that circumscribed the metabolic area was calculated automatically by the dedicated software.

### Statistical analysis

Receiver operating characteristic (ROC) curves were used in order to establish the sensitivity and specificity of the distinct operating points of the four parameters measured before and after nCRT, as well as the differences between the pre- and post-nCRT values, thus allowing the post-nCRT status of the primary tumor site to be classified as a pCR or RD. The result of the histopathological analysis of the surgical specimen was used as the gold standard. The areas under the curve (AUCs) and the corresponding 95% confidence intervals (95% CIs) were used in order to evaluate the accuracy of the four parameters in that classification. Finally, we estimated the sensitivity and specificity of those parameters for the identification of RD when no cancer cells were detected through visual inspection of the primary tumor site after nCRT. Any residual uptake above the background level at the primary tumor site was used as the threshold.

Descriptive statistics—including means and standard deviations; medians and ranges; and absolute and relative frequencies—are presented for some variables. We also present the sensitivity and specificity of the best operation point to classify the patients as achieving a pCR or having RD. Statistical analyses were performed with the Stata software package, version 16.1 (StataCorp, College Station, TX, USA). The level of statistical significance was set at *p* < 0.05.

## RESULTS

### Patient baseline characteristics

Our analysis included 39 patients who underwent ^18^F-FDG-PET/CT before nCRT with a platinum- and taxane-based regimen and between the nCRT and the esophagectomy. The mean time from the pre-treatment ^18^F-FDG-PET/CT to the beginning of the nCRT was 12 ± 6 weeks. The mean time from the last cycle of the nCRT to the post-treatment ^18^F-FDG-PET/CT was 9 ± 4 weeks, and the mean time from the end of nCRT to surgery was 16 ± 6 weeks.

The two chemotherapy regimens adopted were carboplatin plus paclitaxel (in 54% of the patients) and cisplatin plus paclitaxel (in 46%). The radiation doses used were 41.4 cGy (in 54% of the patients), 45.0 cGy (in 28%), and 50.4 cGy (in 18%). The 90-day mortality rate was 15.4% (Table 1).

**Table 1:** Baseline characteristics of the patients included.

Characteristics	(N = 39)
Sex, n (%)	
Male	30 (76.9)
Female	9 (23.1)
Age (years), median (range)	62 (45-76)
Type of esophageal cancer, n (%)	
Squamous cell carcinoma	30 (76.9)
Adenocarcinoma	9 (23.1)
Grade of cellular differentiation, n (%)	
I	3 (7.7)
II	19 (48.7)
III	7 (17.9)
Clinical (pretreatment) stage*, n (%)	
I/II	10 (25.6)
III/IV	29 (74.4)
Presence of RD, n (%)	18 (46.2)
Death within 90 days after surgery, n (%)	6 (15.4)

* In accordance with the 8th edition of the Union for International Cancer Control staging system^(20)^.

### Pathological response

**Figure 1:**
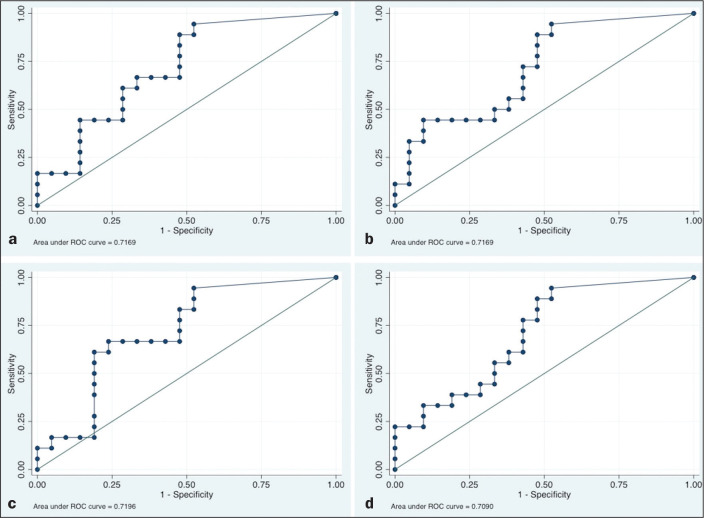
ROC curves of the accuracy of the four ^18^F-FDG-PET/CT parameters after nCRT for the detection of RD, in comparison with the histopathological diagnosis. a: SUV_max_. b: MTV. c: SUV_mean_. d: TLG.

**Figure 2:**
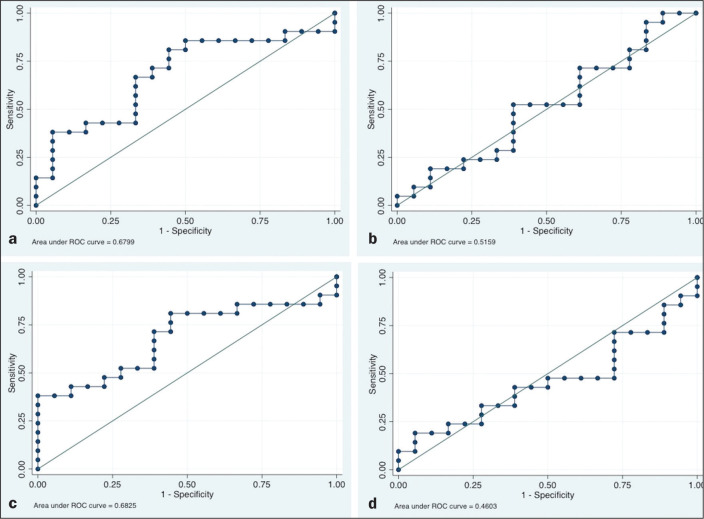
ROC curves of the accuracy of the difference between the pre- and post-treatment values of the four ^18^F-FDG-PET/CT parameters for the detection of RD, in comparison with the histopathological diagnosis. a: SUV_max_. b: MTV. c: SUV_mean_. d: TLG.

The ROC curve analyses of the variables determined by ^18^F-FDG-PET/CT, in terms of their ability to predict RD, are shown in Table 2, as well as in Figures 1 and 2. The pre-treatment values for the ^18^F-FDG-PET/CT parameters were not found to be predictors of RD. For the post-treatment ^18^F-FDG-PET/CT parameters, the AUCs were similar among all four of the variables related to the primary tumor and those AUCs were statistically significant (Figure 1). After nCRT, the AUC was 0.7169 (95% CI: 0.5541-0.8797) for the SUV_max_, 0.7169 (95% CI: 0.5537-0.8801) for the MTV, 0.7196 (95% CI: 0.5544-0.8848) for the SUV_mean_, and 0.709 (95% CI: 0.5449-0.8731) for the TLG. For the difference between the pre- and post-treatment values of the four ^18^F-FDG-PET/CT parameters, only the SUV parameters (SUV_max_ and SUV_mean_) showed significant ability to detect RD (Figure 2). Table 2 summarizes those findings.

**Table 2:** Diagnostic accuracy of the four ^18^F-FDG-PET/CT parameters for the detection of RD, in comparison with the histopathological diagnosis.

^18^F-FDG-PET/CT	Parameter	AUC	SE	95% CI
Before nCRT	SUV_max_	0.4127	0.0955	0.25567-0.57900
	MTV	0.6720	0.0889	0.49783-0.80912
	SUV_mean_	0.4206	0.0951	0.25567-0.57900
	TLG	0.6005	0.0938	0.42100-0.74433
After nCRT	SUV_max_	0.7169	0.0831	0.55411-0.87975
	MTV	0.7169	0.0833	0.55371-0.88015
	SUV_mean_	0.7196	0.0843	0.55436-0.8848
	TLG	0.7090	0.0837	0.54487-0.87312
Difference between pre- and post-nCRT values	SUV_max_	0.6799	0.0891	0.52431-0.82980
	MTV	0.5159	0.0964	0.34780-0.67582
	SUV_mean_	0.6825	0.0884	0.52431-0.82980
	TLG	0.4603	0.0953	0.30095-0.62819

Gray shading indicates variables able to detect RD at the level of significance established.

**Figure 3:**
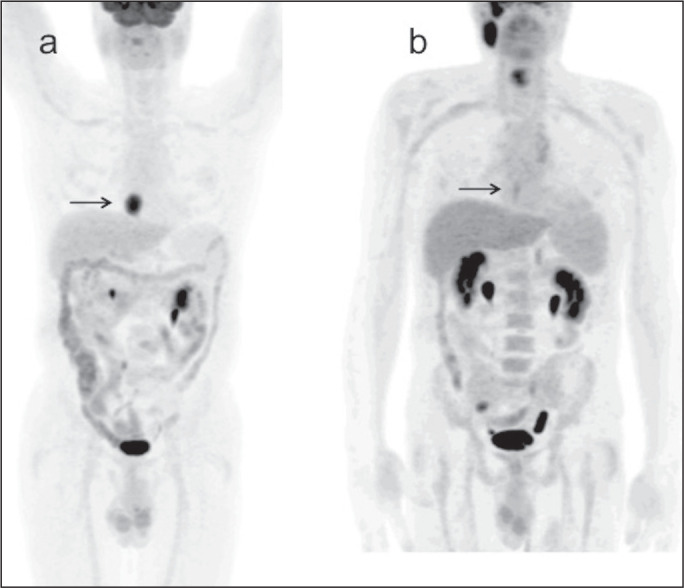
**a**: Maximum intensity projection (MIP) image of the ^18^F-FDG-PET/CT scan acquired before nCRT, showing intense uptake at the primary tumor site in the esophagus (arrow). b: MIP of the ^18^F-FDG-PET/CT scan acquired after nCRT, showing faint uptake at the primary tumor site (arrow). The patient presented RD. There was also nonspecific diffuse uptake in the right masseter and lateral pterygoid muscles, which could be explained by muscle contraction, given that there were no evident anatomical alterations.

**Figure 4:**
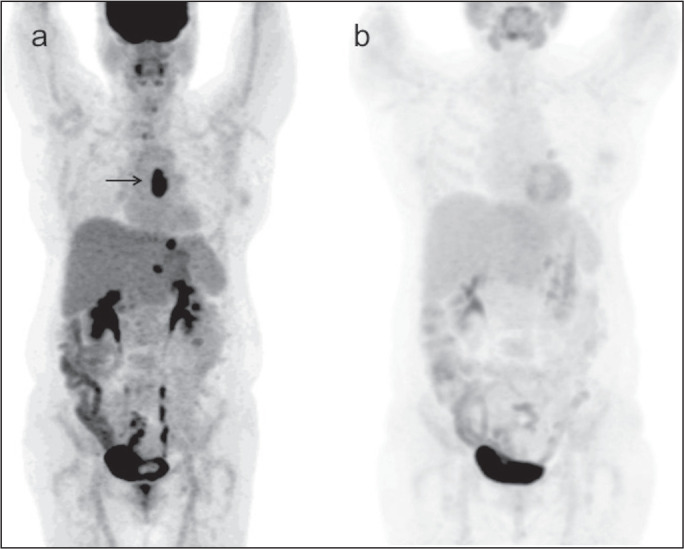
**a**: MIP image of the ^18^F-FDG-PET/CT scan acquired before nCRT, showing intense uptake at the primary tumor site in the esophagus (arrow). b: MIP of the ^18^F-FDG-PET/CT scan acquired after nCRT, showing no detectable uptake at the primary tumor site. The patient presented a pCR.

Given that the main utility of ^18^F-FDG-PET/CT in esophageal cancer is to detect all patients with RD, which improves the selection of surgical candidates, the sensitivity of the modality is more important than is its specificity. Therefore, if the SUV or volumetric parameters of the primary tumor on ^18^F-FDG-PET/CT after nCRT were not “zero” (i.e., the nuclear medicine physician visually detected a region of uptake above the background level at the primary tumor site), the sensitivity for predicting RD was high (94.4%; 95% CI: 72.7-99.9%), although the specificity was intermediate (47.6%; 95% CI: 25.7-70.2%). Figure 3 depicts a patient with residual uptake at the primary tumor site after nCRT. Figure 4 depicts another patient, in whom there was no visually detectable uptake after nCRT. Using any residual uptake above the background level as the threshold, we identified 17 of the 18 patients with RD and 10 of the 21 patients with a pCR (Table 3).

**Table 3:** Contingency table comparing the results of the ^18^F-FDG-PET/CT performed after nCRT therapy with the histopathological results (N = 39). The presence of any uptake above the background level was used as the threshold for the classification of the ^18^F-FDG-PET/CT result as positive for RD.

Post-nCRT result	RD	pCR	n (%)
^18^F-FDG-PET/CT positive	17	11	28 (71.8)
^18^F-FDG-PET/CT negative	1	10	11 (28.2)
n (%)	18 (46.2)	21 (53.8)	39 (100)

## DISCUSSION

In this study of patients with esophageal cancer, we have shown that the ^18^F-FDG-PET/CT parameters obtained for the primary tumor site after nCRT has the potential to predict RD. In this context, the presence of any visually detectable uptake above the background level at the site of the tumor seems to be the best predictor of RD. Our results suggest that some patients without visually detectable uptake on ^18^F-FDG-PET/CT after nCRT would benefit from a watchful waiting approach, somewhat similar to that applied in cases of rectal cancer^(4)^, which avoids the risk of postoperative complications and mortality associated with esophagectomy. Such patients accounted for approximately 28% of our sample. If a watchful waiting approach had been adopted in those cases, the overall mortality rate could have been lower.

Our study has some limitations. First, it was a retrospective, single-center study with small sample size. In addition, although our results provide evidence to support the use of ^18^F-FDG-PET/CT to predict RD after nCRT, the criterion used for ^18^F-FDG-PET/CT classification as positive (any uptake above the background level) is not perfect and failed to detect RD in one of the 18 patients with RD in our sample. Therefore, the method should be used with extreme caution for surgical decision-making, and every choice should be shared with the patient and their family. As a general rule, patients should still be referred for resection. Nevertheless, ^18^F-FDG-PET/CT could be useful in some cases in which surgery is indicated. For example, in patients who deteriorate in the setting of neoadjuvant therapy, who are at higher risk of complications of esophagectomy, and in whom the ^18^F-FDG-PET/CT variables favor the attainment of a pCR, the medical team could offer, in consultation with the patient and their family, the option of adopting a watchful waiting approach.

Despite the fact that the SUV and volumetric parameters are known to be associated with the long-term prognosis in esophageal cancer^(6-13,21)^, there are divergent results in the literature regarding the utility of ^18^F-FDG-PET/CT in predicting the pathological response to nCRT^(11,22,23)^. Most of the relevant studies have used a variety of neoadjuvant regimens, have not assessed th ^18^F-FDG-PET/CT parameters after nCRT, and have not taken a standardized approach to data analysis. Arnett et al.^(22)^ showed that the ^18^F-FDG-PET/CT parameters (SUV_max_, SUV_max_ normalized to liver uptake, and SUV_max_ normalized to blood pool uptake) measured pre- and post-nCRT were not significantly associated with a pCR in a sample of 193 patients, most of whom had adenocarcinoma. However, the authors did not use ROC curves to define a metabolic threshold to separate patients with a pCR from those with RD. In addition, most of the patients in our sample had squamous cell carcinoma, which is known to have a higher probability of a pathological response after chemoradiotherapy, as demonstrated elsewhere^(24)^. That could explain the superiority of our results. In a recent study, Choi et al.^(25)^, analyzing a cohort of patients undergoing trimodal therapy, showed that pre- and post-treatment volumetric parameters from ^18^F-FDG-PET/CT are independent variables associated with a pCR. However, those authors evaluated ^18^F-FDG-PET/CT parameters only as prognostic variables and did not propose the use of ^18^F-FDG-PET/CT as a diagnostic tool for detecting RD. Currently, clinicians lack a reliable tool to facilitate the decision between surgery and a watchful waiting approach after nCRT. Our study provides evidence that ^18^F-FDG-PET/CT could be a useful tool for the detection of RD and has high sensitivity when any residual uptake above the background level at the primary tumor site is used as a threshold.

It should be borne in mind that an inflammatory reaction due to radiation exposure may partially explain the low specificity of ^18^F-FDG uptake in the definition of RD. Previous studies have suggested that an inflammatory response could have a confounding effect on the ^18^F-FDG-PET/CT variables after neoadjuvant therapy^(26,27)^. Therefore, the interval between the end of the nCRT and the post-treatment ^18^F-FDG-PET/CT may be a key factor that could explain some of the variability of the results among the studies and could contribute to the limited specificity. In the present study, the mean time from the last cycle of the nCRT to the post-treatment ^18^F-FDG-PET/CT was relatively short (nine weeks). It is possible that a longer interval would have increased the specificity. Therefore, future studies should attempt to determine whether a longer interval between the last nCRT cycle and ^18^F-FDG-PET/CT could improve the accuracy of the examination in the identification of RD.

It is noteworthy that, in our study, any residual uptake above the background level at the primary tumor site was found to be the best threshold to classify the ^18^F-FDG-PET/CT study as positive or negative for RD. Therefore, the quantitative and semiquantitative parameters (SUV_max_, SUV_mean_, MTV, and TLG) do not appear to be fundamental for the purpose of defining the pathological response, although that should be better analyzed in future studies.

Combining the ^18^F-FDG-PET/CT data with those obtained by other diagnostic methods or with clinical and demographic data could increase the accuracy for the identification of a pCR. In a study of esophageal squamous cell cancer conducted by Molena et al.^(28)^, a ≥ 70% reduction in the SUV_max_ combined with a normal appearance on endoscopy and a lack of RD on biopsy was found to increase the chance of achieving a pCR. Zhang et al.^(29)^ analyzed a model to predict a pCR that combines ^18^F-FDG-PET/CT, clinical data, and demographic features. They found that the model accurately predicted a pCR. Therefore, future analyses of our data are necessary to determine whether combining the results of the ^18^F-FDG-PET/CT study with clinical and demographic parameters, as well as with the results of other diagnostic tests, could further improve the accuracy for the detection of pCR.

The results of the present study do not provide a definitive answer for clinicians who manage esophageal cancer and should be interpreted in the context of the aforementioned limitations. Therefore, larger, controlled prospective studies are warranted in order to determine the true accuracy of ^18^F-FDG-PET/CT for the detection of RD.

## CONCLUSION

The use of ^18^F-FDG-PET/CT after neoadjuvant therapy in patients with esophageal carcinoma has the potential to predict the pathological response. The parameters measured by ^18^F-FDG-PET/CT also facilitate the selection of patients who are eligible for a watchful waiting approach.
